# Emerging roles of Galectin-3 in diabetes and diabetes complications: A snapshot

**DOI:** 10.1007/s11154-021-09704-7

**Published:** 2022-01-27

**Authors:** Yanhua Li, Tian Li, Zhiguang Zhou, Yang Xiao

**Affiliations:** 1 Department of Metabolism and Endocrinology, National Clinical Research Center for Metabolic Diseases, The Second Xiangya Hospital of Central South University, No. 139, Renmin Rd, Changsha, 410011 China; 2 Department of Metabolism and Endocrinology, The Third Hospital of Changsha, 176, West Labour Road, Changsha, 410011 China; 3 School of Basic Medicine, Fourth Military Medical University, No. 169 Changle West Rd, Xi’an, 710032 China

**Keywords:** Galectin-3, Diabetes, Inflammation, Autoimmunity

## Abstract

Galectin-3 is a member of the galectin family, widely expressed in immune cells and plays a role mainly in inflammation, autoimmunity, apoptosis, and chemotaxis. We summarized the roles of Galectin-3 in diabetes and its complications, as well as the underlying mechanisms. Clinical research has determined that the circulating level of Galectin-3 is closely related to diabetes and its complications, thus it is promising to use Galectin-3 as a predictor and biomarker for those diseases. Galectin-3 also may be considered as an ideal therapeutic target, which has broad prospects in the prevention and treatment of diabetes and its complications, especially macrovascular and microvascular complications.

## Introduction

Diabetes mellitus (DM) is a public health problem worldwide. It was reported that the global diabetes prevalence in 2019 was estimated to be 463 million people, rising to 578 million by 2030 and 700 million by 2045 [1–4]. Meta-inflammation theory suggests that both type 1 diabetes mellitus (T1D) and type 2 diabetes mellitus (T2D) are chronic inflammatory diseases. T1D involves inflammation in pancreatic islet tissue, while inflammation in adipose tissue result in T2D, which are attributed to the imbalance of pro-inflammatory and anti-inflammatory cells and molecules [5, 6].

Galectin-3, encoded by LGALS3 gene, is a member of β-galactoside-binding lectin family subtype of galectin, formerly known as carbohydrate-binding protein-35 (CBP-35) [7–9]. Human Galectin-3 is a 26 kDa size lectin, mainly comprises a C-terminal carbohydrate recognition binding domain (CRD) and N-terminal domain [10–12]. Galectin-3 is widely distributed in the tissues such as human hematopoietic tissues, thymus, lymph nodes, spleen, and mainly produced by immune cells such as macrophages, mast cells, and eosinophils, et al., and can be secreted extracellularly [13, 14]. It is regarded as a powerful pro-inflammatory signaling factor, which plays an important role in the activation, chemotaxis, and cytokine release of inflammatory cells. Increasing evidence has proven that levels of circulating Galectin-3 are elevated in chronic inflammatory diseases including obesity, diabetes and its complications, suggesting that Galectin-3 is closely related to those disease status [15]. Thus, clarifying the potential pathogenic mechanism of Galectin-3 in diabetes and diabetic complications will provide a basis for finding new biomarkers for precision disease prediction and early-diagnosis, and developing potential therapeutic targets.

Therefore, we reviewed the clinical evidence of Galectin-3 in diabetes and its complications and discussed the potential mechanism of such relevance. Furthermore, further perspectives and potential directions were provided, which can be used as a reference for further clinical research and scientific foundation for new drug development.

## Galectin-3 in type 2 diabetes

T2D is a chronic inflammatory disease, characterized by obesity-associated insulin resistance [16–20]. Autoimmunity plays a pivotal part in a series of chronic diseases [21–23]. Several clinical studies reported that circulating level of Galectin-3 was significantly higher in T2D patients [24–28] (Table 1). Weigert et al. [24] found that serum Galectin-3 level was significantly higher in T2D patients. Interestingly, they reported that Galectin-3 level reduced by metformin treatment, the mechanism of which may be metformin lowered oxidative stress and the formation of advanced glycation end products (AGEs), and then decreased Galectin-3 expression. In 2019, a cross-sectional study conducted by Atalar et al. [25] enrolling healthy control, prediabetes and T2D, found that serum Galectin-3 increased in T2D but not prediabetes and healthy controls, and had a positive correlation with FPG and HbA1c, as well as hs-CRP, whose level was a useful biomarker for early estimating the chronic inflammatory process in diabetes. The findings of this study suggested that Galectin-3 may play a role in the progression of prediabetes stage to diabetes stage. In summary, all current human studies provided strong evidence of Galectin-3 as a marker, even a pathogenic factor, of T2D.

**Table 1:** Changes in the galectin-3 levels in diabetes and complications in human

**Authors, Year**	**Type of Study**	**Study Subjects**	**Major Findings**
**Type 1 diabetes**			
Karlsen et al. 2006 [40]	Genetic research	Non-diseased offspring of 257 T1D families	Galectin-3 was a susceptibility gene for T1D
**Type 2 diabetes**			
Weigert et al. 2010 [24]	Cross-sectional study	23 normal-weight controls, 30 overweight controls, 30 T2D patients (Male)	Galectin-3 was increased in overweight controls and T2D
Lin et al. 2021[26]	Cross-sectional study	270 controls, 135 T2D patients	Galectin-3 was increased in T2D
Ohkura et al. 2014 [27]	Cross-sectional study	20 T2D patients	Low serum Galectin-3 concentrations strongly correlated with insulin resistance and hyperinsulinemia
Atalar et al. 2019 [25]	Cross-sectional study	41 controls, 34 prediabetes, 84 T2D patients	Galectin-3 was increased in T2D
Vora et al. 2019 [28]	Multiethnic cohort study	6586 participants from the Dallas Heart Study	Galectin-3 was associated with diabetes prevalence and incidence
**Diabetic macrovascular complication**			
Seferovic et al. 2014 [81]	Cross-sectional study	189 participants (70 T2D, 60 T2D with hypertension, 71 hypertention)	Galectin-3 was increased in T2D with hypertention
Ozturk et al. 2015 [63]	Observational study	157 DM patients (80 non-coronary artery disease, 77 coronary artery disease)	Galectin-3 was increased in CAD group
Saeed et al. 2021 ([86])	Cohort study	295 T1D subjects	Galectin-3 was significantly associated with future CHD in subjects with type 1 diabetes
**Diabetic nephropathy**			
Tan et al. 2018 [67]	Prospective cohort study	1320 T2D participants	Galectin-3 was independently associated with the doubling of serum creatinine and incident macroalbuminuria
Hodeib et al. 2019 [68]	Single-center prospective cohort study	100 T2D with ACR < 30 mg/g; 100 T2D with ACR within 30–300 mg/g; 100 T2D with ACR > 300 mg/g	Galectin-3 was increased in patients with ACR > 300 mg/g
Iacoviello et al. 2016 [87]	Cross-sectional study	61 individuals with microalbuminuria; 133 with normoalbuminuria	Galectin-3 was increased in microalbuminuria
**Diabetic foot**			
Gunes et al. 2018 [77]	Prospective cohort study	91 participants (30 controls; 30 T2D; 31 T2D with DFU)	Galectin-3 was correlated with the VEGF-A level
**Diabetic cardiomyopathy**			
Bolotskykh and Rudyk 2014 [82]	Cross-sectional study	74 patients with HFpEF with and without T2D	Galectin-3 was increased in heart failure patients

Efforts on mechanism investigation also have been made both in cell studies and animal studies. Li et al. [29] showed that the genetic depletion of Galectin-3 improved both systemic and tissue insulin sensitivity, and glucose tolerance in HFD mice, indicating Galectin-3 induces insulin resistance and deteriorates glucose homeostasis. Mechanically, they found that elevated circulating Galectin-3 from macrophages directly interacted with insulin receptor, impaired the major steps of insulin signaling pathway, inducing cellular insulin resistance in adipose tissue, liver and muscle. Petrovic et al. [30] further demonstrated that pancreatic Galectin-3 overexpression promoted β-cell apoptosis triggered by cytokines and palmitate, and increased NO_2_-induced oxidative stress in β cells, suggesting that Galectin-3 participants in β-cell damage and insulitis in HFD-induced T2D. Yu et al. [31] reported that the inhibition of Galectin-3 by TD139, an effective inhibitor of the galactoside binding pocket of Galectin-3, improved insulin resistance in HFD-induced mice, suggesting the potential of TD139 in the treatment of T2D.

In conclusion, serum Galectin-3 is elevated in T2D patients, and the elevated Galectin-3 plays a role in pathogenesis of T2D by disturbing insulin signaling pathway in insulin-targeting organs and inducing β-cell inflammation and death in pancreatic islets. Thus, it is promising to use Galectin-3 as a predictive biomarker and therapeutic target for T2D.

## Galectin-3 in type 1 diabetes

In recent years, the incidence of T1D has increased rapidly at a rate of 3%-5% per year worldwide. T1D is an autoimmune disease caused by the selective destruction of insulin-producing β cells. Damaged β cells release their own antigens and present them to autoreactive T cells in the pancreatic draining lymph nodes (PLN) [32, 33]. These activated autoreactive T cells migrate to the pancreatic islets, thereby promoting inflammation and mediating β-cell apoptosis [34–36].

Early studies have shown that Galectin-3 plays a pivotal role in autoimmune diseases including autoimmune encephalomyelitis (EAE) [37], rheumatoid arthritis (RA) [38] and systemic sclerosis [39], et al. Karlsen, A.E. et al. identified six polymorphisms in the Galectin-3 gene (LGALS3) and proved that a haplotype containing three SNPs transmitted to unaffected offspring in 257 T1D families increased significantly. This finding was verified in an independent set of 170 T1D families, suggesting that LGALS3 is a T1D susceptibility gene, providing genetic association between Galectin-3 and T1D [40].

The destruction of pancreatic β-cells in T1D is mainly due to disorders of adaptive immune cells as well as innate immune cells [41]. Macrophage is the first-line cell to invade the pancreatic islets, and acts as an antigen presenting cell and an effector cell, contributing to the pathogenesis and development of autoimmune diabetes [42–45]. A study *in vitro* showed that Galectin-3 chemoattracts monocytes and macrophages through the G protein coupling pathway [46]. Galectin-3 involves in the migration of neutrophils to sites of infection or inflammation, activates neutrophils and promotes their adhesion [47, 48]. If the infection persists and is not controlled by neutrophils, Galectin-3 recruits macrophages to the site of infection, strengthening further defenses [49, 50]. Galectin-3 also participates in the innate immune response and is considered to be a regulator of T cell activation [51], thus promoting pro-inflammatory cytokines which up-regulate the apoptotic signal [52, 53]. Cytokines can increase the expression of Galectin-3 mRNA, making it a vicious circle process [54].

*In vitro* data provided that the expression of Galectin-3 was upregulated in BB-DP islets after IL-1β exposure, however, whether Galectin-3 elevation is a causal factor leading to apoptosis, or a compensatory effect to combat apoptosis is unclear [55]. Saksida et al. [52] proved that genetic deletion or pharmacological inhibition of Galectin-3 preserved β cell function and promoted survival by reducing mitochondrial apoptotic pathway triggered by pro-inflammatory cytokines, indicating the pro-inflammatory property of Galectin-3 in pancreatic β cell apoptosis. The main mechanism is that Galectin-3 downregulates the expression of major genes in this apoptotic pathway and upregulates the expression of anti-apoptotic genes [52, 55]. By analyzing TD139-treated RINm5F cells, TD139 significantly ameliorates cell apoptosis by Galectin-3 under the action of various cytokines [56]. In contrast, an *in vitro* study showed that overexpression of Galectin-3 in RIN-cell inhibited cytokine mediated apoptosis, suggesting the protective role of Galectin-3 against cytokine toxicity induced β cell apoptosis [40]. Indeed, different location of Galectin-3 exerts various functions, for example, cytoplasmic Galectin-3 has anti-apoptotic activity maintaining mitochondrial integrity, whereas nuclear and extracellular Galectin-3 plays pro-apoptotic role [57], which may explain the controversial results.

Animal studies also conducted to investigate whether Galectin-3 contributes to the pathogenesis of T1D. Mensah-Brown et al. demonstrated that the lack of Galectin-3 protected C57BL/6 mice from MLD-STZ induced diabetes, accompanied with significant less mononuclear infiltration in the pancreatic islets and with retention of higher insulin content when compared with WT mice. Macrophages in the abdominal cavity and pancreatic draining lymph nodes from Galectin-3 knockout mice expressed lower interferon-γ (IFN-γ), TNF-α, IL-17 and inducible nitric oxide synthesis (iNOS) than those from WT mice [58]. On summary, the studies above confirmed that both the deletion of Galectin-3 gene or pharmacological inhibition reduces the apoptosis of pancreatic β-cells. Therefore, Galectin-3 plays a role in the occurrence of β-cell destruction in T1D and is a promising therapeutic target for T1D patients.

## Galectin-3 in diabetes complications

The most common complication of diabetes is vascular disease, which is also the main cause of morbidity and mortality in diabetic patients [59]. Vascular complications are mainly divided into macrovascular complications including cerebrovascular, cardiovascular and peripheral vascular disease, and microvascular complications including retinopathy, nephropathy, and diabetic foot [60].

### Macrovascular complications

The major vascular complication of diabetes is the accelerated formation of atherosclerosis, which involves important blood vessels in the body, such as coronary arteries, carotid arteries, and peripheral arteries [61]. Galectin-3 plays a pro-inflammatory role in the occurrence and development of atherosclerosis and can be used as a risk factor for atherosclerosis [62].

Ozturk et al. performed coronary CT scans on 158 patients with T2D and reported that Galectin-3 was positively correlated with the number of coronary arteries with atherosclerosis [63]. Saeed et al. followed up a population-based nationwide cohort in Norway, and found that after the adjustment of conventional risk factors, Galectin 3 was significantly associated with the risk of future coronary heart disease (CHD) and may help to predict the occurrence of CHD in patients with T1D [64]. These findings suggest that Galectin-3 could be a predictor for coronary atherosclerosis in patients with T1D or T2D.

### Microvascular complications

#### Diabetic nephropathy

Diabetic nephropathy is the main cause of end-stage renal disease in diabetic patients [65, 66]. A prospective study involving 1320 cases of T2D with an average follow-up of 9 years showed that the level of serum Galectin-3 was independently associated with the progression of diabetic nephropathy [67]. Compared with patients with baseline serum Galectin-3 levels in the lower quarter, patients with Galectin-3 levels in the upper quarter had a three-fold increase in the risk of renal function decline and a two-fold increase in the risk of massive proteinuria [67]. The results of a single-center prospective clinical study demonstrated that Galectin-3 levels are elevated in diabetic nephropathy and are positively correlated with the urine albumin/creatinine ratio [68]. These findings suggest that increased circulating Galectin-3 may be predictive of poor prognosis in diabetic nephropathy.

#### Diabetic retinopathy

Approximately two-thirds of diabetic patients have diabetic retinopathy. Studies have found that Galectin-3 is related to the pathogenesis of diabetic retinopathy. Galectin-3 may affect visual function during diabetes by combining with AGEs [69]. An animal study by Bauer et al. on lipopolysaccharide-induced neuroinflammation found that Galectin-3 is expressed in both Müller cells and microglia/macrophages of the normal retina; and demonstrated that increase in Galectin-3 expression was accompanied by significant increased neurotoxicity in the explanted retina [70]. Abreu et al. showed that the damage to optic nerve fibers caused by microglia/macrophages was reduced in Galectin-3 knockout mice [71]. Compared with the microglia of wild-type mice, the microglia of Galectin-3 knockout mice were less pro-inflammatory, resulting in excess white matter [72] and reduced retinal ganglion cell apoptosis [71]. The cause may be that iNOS is less activated in the optic nerve of Galectin-3 knockout mice [73]. Stitt et al. [74] infused preformed AGEs into wild-type and Galectin-3 knockout mice and found that, in oxygen-induced proliferative retinopathy, Galectin-3 knockout mice have reduced retinal ischemia and higher retinal angiogenesis potential. Therefore, Galectin-3 seems to be a pivotal molecule triggering neurodegenerative, oxidative and inflammatory processes preceding vascular modifications in diabetic retinopathy, and its modulation might be a useful tool to prevent diabetic visual complications.

#### Diabetic foot

Diabetic foot is one of the long-term complications of diabetes, and 50%-60% of diabetic foot patients have some degree of peripheral artery disease [75, 76]. Studies have reported that Galectin-3 is associated with the occurrence of diabetic foot ulcers [75, 77]. Galectin-3 promotes blood vessel formation and new blood vessel formation because Galectin-3 increases the level of VEGF-A [78, 79]; thus, Galectin-3 promotes the healing of diabetic foot ulcers (DFUs). In a prospective study with 30 healthy controls, 30 patients diagnosed with T2D but without DFUs and evident peripheral artery disease (PAD) as the diabetic control group and 31 patients diagnosed with T2D with DFUs at Wagner stage 2–4, serum Galectin-3 levels together with VEGF-A were significantly higher in T2D with DFUs compared with the other two groups, indicating as a defense mechanism against DFUs, thus contributing to wound healing [77]. When chronic skin damage occurs, Galectin-3 expression is inversely correlated with the level of AGEs in diabetic or non-diabetic patients, suggesting that Galectin-3 helps to reduce the accumulation of AGEs in the skin wound [80]. These studies provide an insight about Galectin-3 as an emerging biomarker which may give an indication of prognosis in diabetic foot with special reference to DFUs.

### Diabetic cardiomyopathy

Diabetic cardiomyopathy is the main cause of heart failure in diabetes patients. Jelena et al. enrolled 189 participants with T2D and/or arterial hypertension (HT), and found that levels of galectin-3 were higher in patients with both T2D and HT, and correlated with left ventricular (LV) mass, indicating the potential role of Galectin-3 for early detection of myocardial structural and functional alterations [81]. Bolotskykh et al. found that non-diabetic patients with heart failure are accompanied by insulin resistance, and the levels of Galectin-3, TNF-α and insulin in such heart failure patients are significantly increased [82]. Myocardial inflammation and fibrosis are accompanied by changes in the Galectin-3 concentration [83]. Further studies confirmed that the mechanism of inducing cardiomyocyte changes including hypertrophy, fibrosis, increased stiffness, and impaired relaxation is triggered by the pro-inflammatory environment caused by adipose tissue dysfunction, and Galectin-3 can be used as a biomarker for changes in myocardial function and fibrosis caused by high calories [84, 85]. This finding shows that Galectin-3 is involved in the development of cardiac fibrosis and impaired myocardium remodeling, resulting in heart failure and atrial fibrillation [85]. The role of Galectin-3 as a prognostic marker of heart failure is described, and the tentative use of Galectin-3 inhibition is a potential therapeutic approach to prevent cardiac inflammation and fibrosis.

## Further perspectives

**Fig. 1:**
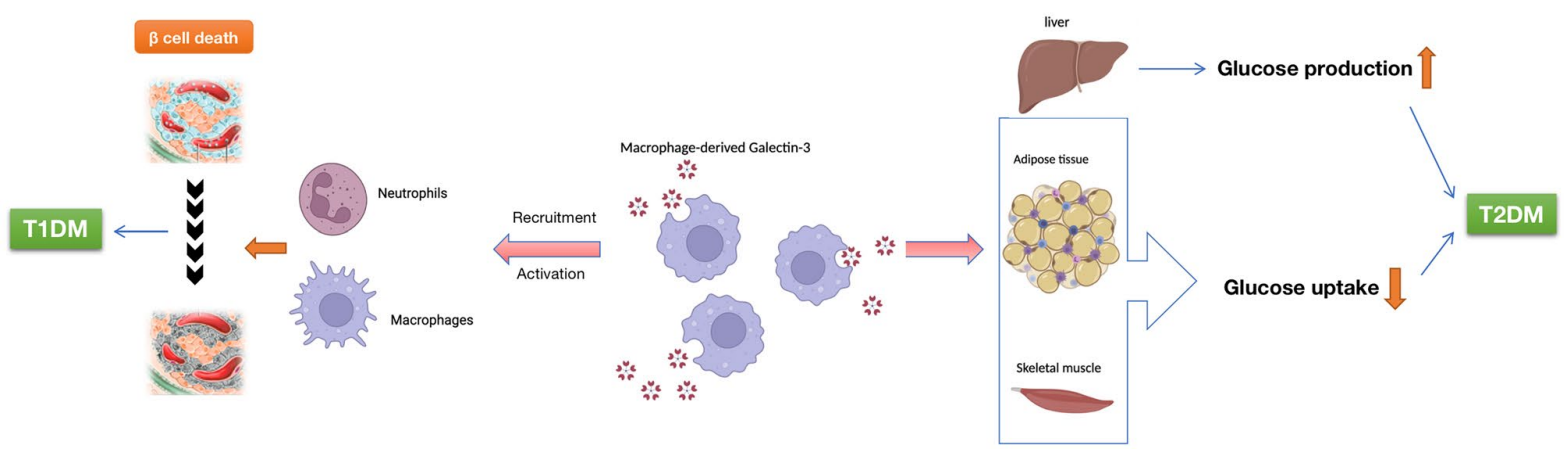
The role of galectin-3 in diabetes. In type 1 diabetes, macrophage-derived Galectin-3 participates in β cell death by recruiting and activating innate immune cells, including neutrophils and macrophages, to islets; In type 2 diabetes, Galectin-3 directly interacted with insulin receptor, impaired the major steps of insulin signaling pathway, inducing cellular insulin resistance in adipose tissue, liver and muscle

As a powerful cytokine related to inflammation, autoimmunity, apoptosis, and chemotaxis, Galectin-3 is involved in several metabolic abnormalities, mainly in diabetes and diabetic complications (Fig. 1). A large number of clinical studies have shown that Galectin-3 is related to the occurrence of diabetes and its complications. The main conclusion is that the level of serum Galectin-3 is higher in patients with diabetes and its complications.

There are also a large number of animal experiments revealing that Galectin-3 promotes the inflammation of pancreatic islet β cells and insulin target organs, leading to pancreatic β cell failure and insulin resistance, which in turn leads to diabetes. According to existing evidence, Galectin-3 inhibitors such as TD139 can reduce the expression of Galectin-3, thereby improving diabetes and its complications. However, whether Galectin-3 can be used in clinical medicine still needs to be resolved.

To fill in the current gaps, the following research including: 1) To further clarify the specific mechanism of Galectin-3 in diabetes and remove obstacles for clinical research. 2) To develop safe and effective Galectin-3 inhibitors for the prevention and treatment of diabetes are warrant.

## Abbreviations

AGEs: Advanced glycation end products
BMI: Body mass index
CEA: Carcinoembryonic antigen
CHD: Coronary heart disease
CPB-35: Carbohydrate-binding protein-35
CRD: Carbohydrate recognition binding domain
CRP: C reactive protein
CVD: Cardiovascular diseases
DFUs: Diabetic foot ulcers
DM: Diabetes mellitus
DXA: Dual-energy x-ray absorptiometry
HFD: High fat diet
IRS1: Insulin receptor substrate 1
JNK: C-Jun-N-terminal kinase
NASH: Nonalcoholic steatohepatitis
NLRP3: NOD-like receptor family pyrin domain protein 3
LAMP-1: Lysosomal-associated membrane proteins 1
LAMP-2: Lysosomal-associated membrane proteins 2
LncRNA: Long non-coding RNA
LPS: Lipopolysaccharide
OGTT: Oral glucose tolerance test
PAD: Peripheral artery disease
PPAR: Peroxisome proliferator-activated receptor
T1D: Type 1 diabetes mellitus
T2D: Type 2 diabetes mellitus
